# The Polymorphisms of Oligonucleotide Probes in Wheat Cultivars Determined by ND-FISH

**DOI:** 10.3390/molecules24061126

**Published:** 2019-03-21

**Authors:** Tianheng Ren, Maojie He, Zixin Sun, Feiquan Tan, Peigao Luo, Zongxiang Tang, Shulan Fu, Benju Yan, Zhenglong Ren, Zhi Li

**Affiliations:** 1 College of Agronomy, Sichuan Agricultural University, Wenjiang, Chengdu 611130, Sichuan, China; szx080598@163.com (Z.S.); tanfq@sicau.edu.cn (F.T.); lpglab@sicau.edu.cn (P.L.); zxtang@sicau.edu.cn (Z.T.); fusl@sina.com (S.F.); renzllab@sicau.edu.cn (Z.R.); 2 Provincial Key Laboratory for Plant Genetics and Breeding, Wenjiang, Chengdu 611130, Sichuan, China; hemaojieo@163.com (M.H.); yanbenju@sicau.edu.cn (B.Y.); 3 College of Life Science, Sichuan Agricultural University, Ya’an 625014, Sichuan, China

**Keywords:** ND-FISH, Oligo-pTa535-1, Oligo-pSc119.2-1, chromosome, wheat, mutant

## Abstract

Non-denaturing fluorescence in situ hybridization (ND-FISH) has been used to distinguish wheat chromosomes and to detect alien chromosomes in the wheat genome. In this study, five different oligonucleotide probes were used with ND-FISH to examine 21 wheat cultivars and lines. These oligonucleotide probes distinguished 42 wheat chromosomes and also detected rye chromatin in the wheat genome. Moreover, the signal patterns of the oligonucleotide probes Oligo-pTa535-1 and Oligo-pSc119.2-1 showed high polymorphism in the wheat chromosomes. A total of 17.6% of the A group chromosomes, 25.9% of the B group chromosomes and 8.9% of the D group chromosomes showed obvious mutations when they were compared to the standard ND-FISH signal patterns, and most of them were Oligo-pSc119.2-1 mutants. The results suggested that these polymorphisms could be induced by the crossing of wheat cultivars. The results provided more information for the further application of oligonucleotide probes and ND-FISH.

## 1. Introduction

*In situ* hybridization (ISH) is a molecular, cytogenetic technique that uses labeled DNA or RNA probes to directly target specific nucleic acid sequences on chromosomes [1,2]. It can directly locate probes on specific chromosome arms and detect sequences in the genome that are homologous to the corresponding probes. Fluorescence in situ hybridization (FISH) is the most common type of ISH, and uses different probes labeled with different colors of fluorescein to analyze different target DNA sequences at one time [3,4,5]. The widespread and specific distribution of tandem repeats in the genome enables them to serve as important DNA molecular markers in the genome, as well as cytological probes [6,7]. The repetitive sequences pSc119.2, pTa-535, pAs1, and pTa71 are usually used as probes in FISH analyses to distinguish between wheat and rye chromosomes [1,5,8,9,10].

Currently, a new, simple and precise ISH technique named non-denaturing fluorescence in situ hybridization (ND-FISH) has been developed [9,11,12,13,14,15,16]. The main advantages of ND-FISH are as follows: It does not require the preparation of probes which can be synthesized commercially, there is no denaturation process and a short hybridization time, and it has a lower cost than FISH [13,14]. Based on these advantages, this technique has been widely used to identify wheat and chromosomes from alien species [9,13,14,15,16]. The design of the oligonucleotide probes is the key to accurately identifying chromosomes through ND-FISH [15]. The oligonucleotide probes, based on tandem repeat sequences, can easily identify single wheat chromosomes. For example, the tandem repeat sequence pSc119.2, which was cloned from the *Secale cereale* genome, is mainly distributed on all rye chromosomes and on the B genome chromosomes of wheat [5,9,17], and the oligonucleotide probe Oligo-pSc119.2 can replace the tandem repeat sequence pSc119.2 to distinguish wheat and rye chromosomes [9,14,18]. The tandem repeat sequence pTa-535 could also be replaced by the oligonucleotide probe Oligo-pTa535 to distinguish different wheat chromosomes [9,14,18,19]. The oligonucleotide probes can distinguish, not only the wheat chromosomes, but also the chromosomes of wheat relatives. For example, the oligonucleotide probe Oligo-1162 or Oligo-Ku combined with Oligo-pSc200 and Oligo-pSc250 can detect rye chromosomes in wheat genetic backgrounds [14,15]. In addition, oligonucleotide probes can detect centromeric regions. For example, Oligo-pAWRC.1 can detect the centromeric regions of rye chromosomes with intensity signal patterns, and Oligo-CCS1 can detect the centromeric regions of both rye and wheat chromosomes [9,13].

These probes sometimes show different signal patterns in different plant materials due to changes in the copy number and location of the tandem repeat sequences or the corresponding position of the oligonucleotides on the chromosomes. The signal patterns of pSc119.2 were mutated on the 2A, 4A, 5A and 3B chromosomes in the wheat-rye 1RS.1BL translocation line RT1104 [20]. The signal patterns of pSc119.2 were also different on the 4A and 5A chromosomes in another 1RS.1BL translocation line RT828 [5]. Oligo-s120.2 and Oligo-s120.3 showed polymorphisms of their signal patterns on the 1B and 6B chromosomes of the Chinese spring, Miangyang11 and Chuan-nong27 cultivars [21]. In addition, Oligo-k566 also showed polymorphisms of signal patterns on the 5B chromosome of these three common wheat varieties [21]. These probes can be used to study the chromosomal polymorphisms of different wheat cultivars and hybrid offspring of wheat and related species with different signal patterns on the same chromosomes, which is potentially of value for wheat breeding programs.

Common wheat (*Triticum aestivum* L.) is an allohexaploid (AABBDD, 2*n* = 42) and is one of the most important crops in the world. The A-, B-, and D-genome of wheat was generally assumed to have originated from *Triticum boeoticum* (AA, 2*n* = 14), *Aegilops speltoides* (BB, 2*n* = 14) and *Aegilops tauschii* (DD, 2*n* = 14), respectively [22]. The repeat sequences copies are as high as 60%–80% in wheat genome. Therefore, the FISH or ND-FISH techniques which were founded on the bases of repeat sequences were very suitable for wheat chromosome identification. However, the polymorphism of the most important and widely used oligonucleotide probes, Oligo-pSc119.2-1 and Oligo-pTa535-1, and the polymorphisms of the signal patterns between wheat cultivars or their wheat parents have not been reported. In the present study, 21 wheat cultivars and several of their wheat parents were analyzed by ND-FISH. The polymorphisms of the signal patterns among, not only different plant materials, but also cultivars that have the same parents and between the offspring and parents were revealed. In addition, the “Chuan-nong” series of wheat cultivars are the most important cultivars in Southwestern China. Chromosomal analysis of these materials will provide more information for wheat breeding programs.

## 2. Results

### 2.1. The ND-FISH Signals Indicate Polymorphisms in A-Genome Chromosomes

**Figure 1:**
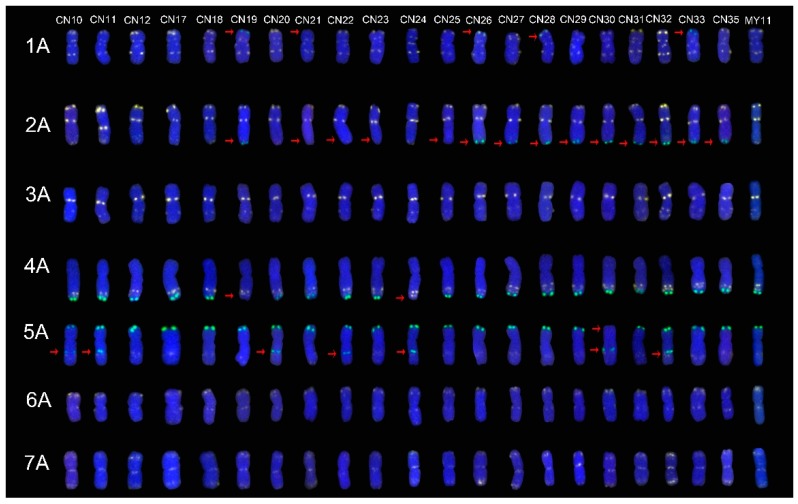
The ND-FISH (non-denaturing fluorescence in situ hybridization) results of A-genome chromosomes of all materials. From left to right: CN10, CN11, CN12, CN17, CN18, CN19, CN20, CN21, CN22, CN23, CN24, CN25, CN26, CN27, CN28, CN29, CN30, CN31, CN32, CN33 and CN35, respectively. From top to bottom: 1A, 2A, 3A, 4A, 5A, 6A and 7A chromosome, respectively. The red arrows showed the mutant signal patterns on chromosomes. Yellow: Oligo-pTa535-1; Green: Oligo-pSc119.2-1; Red: Oligo-Ku, Oligo-pSc200 and Oligo-pSc250; Blue: DAPI.

The ND-FISH signal patterns in chromosomes from different plant materials showed high polymorphism. The signal from Oligo-pTa535-1 on the 1A short arm was absent in CN19, CN21 and CN33 cultivars. However, an additional signal from Oligo-pSc119.2-1 on the 1A short arm was present in CN19, CN26, CN28 and CN33 cultivars (Figure 1). In the 2A chromosomes, the signal from Oligo-pTa535-1 on the 2A long arm was missing in CN21, CN22, CN23, CN25 cultivars, and an additional signal from Oligo-pSc119.2-1 was present on the 2A long arm in CN19, CN26, CN27, CN28, CN29, CN30, CN31, CN32, CN33 and CN35 cultivars (Figure 1). The signal from Oligo-pSc119.2-1 was absent on the 4A long arm in CN19 and CN24 cultivars. An additional signal from Oligo-pSc119.2-1 was present on the 5A long arm in CN10, CN11, CN20, CN22, CN24, CN30 and CN32 cultivars, but the signal from Oligo-pSc119.2-1 on the 5A short arm was absent in the CN30 cultivar (Figure 1). Although there were no obvious mutations in the 3A, 6A and 7A chromosomes, the strong signal from Oligo-pTa535-1 was different from the standard signal from Oligo-pTa535-1 in the MY11 cultivar. For example, the CN26 cultivar showed an intense signal pattern from Oligo-pTa535-1 on the terminus of the 7A long arm (Figure 1).

### 2.2. The ND-FISH Signals Indicate Polymorphisms in B-Genome Chromosomes

**Figure 2:**
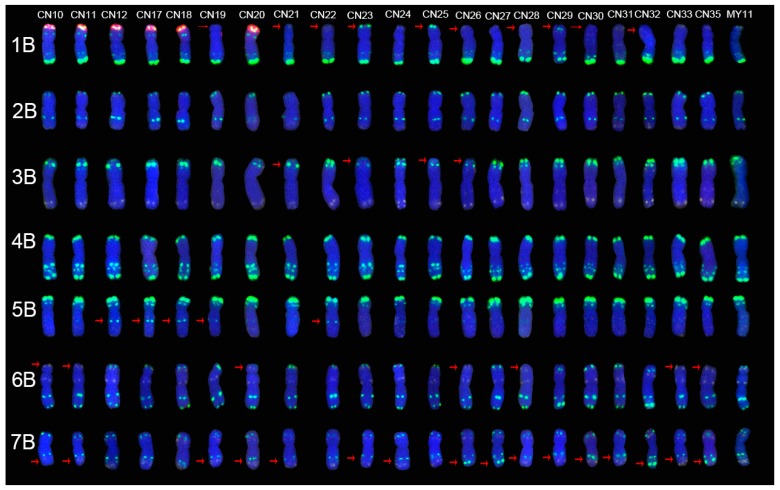
The ND-FISH results of B-genome chromosomes of all materials. From left to right: CN10, CN11, CN12, CN17, CN18, CN19, CN20, CN21, CN22, CN23, CN24, CN25, CN26, CN27, CN28, CN29, CN30, CN31, CN32, CN33 and CN35, respectively. From top to bottom: 1B, 2B, 3B, 4B, 5B, 6B and 7B chromosome, respectively. The red arrows showed the mutant signal patterns on chromosomes. Yellow: Oligo-pTa535-1; Green: Oligo-pSc119.2-1; Red: Oligo-Ku, Oligo-pSc200 and Oligo-pSc250.Blue: DAPI.

The results showed that CN10, CN11, CN12, CN17, CN18 and CN20 cultivars harbor a pair of 1RS.1BL translocation chromosomes. These chromosomes are the 1RS chromosome arm of rye replaced the 1BS chromosome arm of wheat (Figure 2). The signal from Oligo-pTa535-1 on the 1B short arm was missing in CN19, CN21, CN22, CN26, CN28, CN30 and CN32 cultivars. The signal from Oligo-pTa535-1 on the 1B short arm was instead replaced with the signal from Oligo-pSc119.2-1 in CN23, CN25 and CN29 cultivars (Figure 2). The signal from Oligo-pSc119.2-1 on the terminus of the 3B short arm was much weaker in CN21, CN23, CN25 and CN26 cultivars than of those in the 3B chromosome of the MY11 and other cultivars. The signal from Oligo-pSc119.2-1 on the 5B long arm was obviously stronger in CN12, CN17, CN18, CN19 and CN22 cultivars than in other cultivars (Figure 2). The signal from Oligo-pSc119.2-1 on the 6B short arm was absent in CN10, CN11, CN20, CN26, CN28, CN33 and CN35 cultivars. The signal from Oligo-pTa535-1 on the 7B long arm in the CN10 cultivar was weaker than those in other cultivars. The signal from Oligo-pSc119.2-1 on the subtelomere of the 7B long arm was missing in the CN11, CN19, CN20, CN21, CN23, CN24, CN28, CN29, CN30, CN31 and CN33 cultivars. In contrast, this signal was much stronger in CN26, CN27, CN32 and CN35 cultivars (Figure 2).

### 2.3. The ND-FISH Signals Indicate Polymorphisms in D-Genome Chromosomes

**Figure 3:**
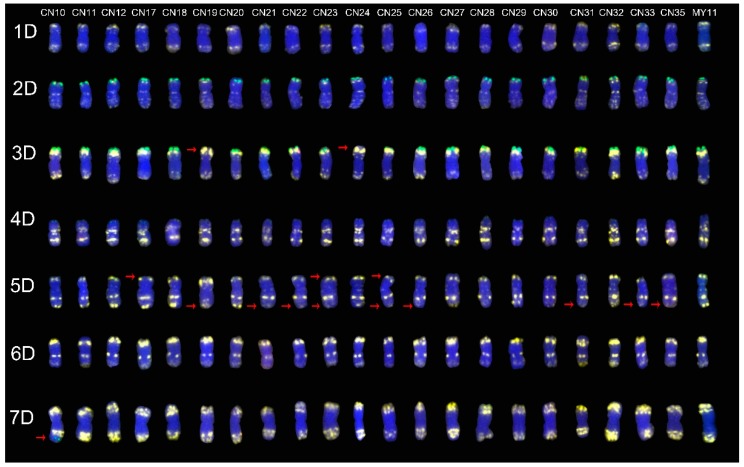
The ND-FISH results of D-genome chromosomes of all materials. From left to right: CN10, CN11, CN12, CN17, CN18, CN19, CN20, CN21, CN22, CN23, CN24, CN25, CN26, CN27, CN28, CN29, CN30, CN31, CN32, CN33 and CN35, respectively. From top to bottom: 1D, 2D, 3D, 4D, 5D, 6D and 7D chromosome, respectively. The red arrows showed the mutant signal patterns on chromosomes. Yellow: Oligo-pTa535-1; Green: Oligo-pSc119.2-1; Red: Oligo-Ku, Oligo-pSc200 and Oligo-pSc250.Blue: DAPI.

The signal from Oligo-pSc119.2-1 on the 3D short arm in CN19 and CN24 cultivars was absent. The signal from Oligo-pSc119.2-1 on the 5D short arm was absent in CN17, CN23 and CN25 cultivars. Moreover, the strong signal from Oligo-pTa535-1 on the 5D long arm was missing in CN19, CN21, CN22, CN23, CN25, CN26, CN31, CN33 and CN35 cultivars. The signal from Oligo-pTa535-1 on the terminus of the 7D long arm was replaced by the signal from Oligo-pSc119.2-1 in the CN10 cultivar (Figure 3). Like the group A chromosomes, the signals still showed different intensity patterns (Figure 3). This was despite the fact that there were no obvious mutations in the signal from Oligo-pTa535-1 on many chromosomes in the D-genome chromosomes.

### 2.4. The Mutant Rate of Signal Patterns of ND-FISH

A total of 88 mutated signal patterns were distributed on 79 chromosomes (17.9%, 79/441). They were different when compared with the standard signal patterns of the MY11 cultivar. Among them, 26 mutants are in the A chromosome group (17.6%, 26/147), 40 are in the B chromosome group (27.2%, 40/147, not including the 1RS chromosome), and 13 are in the D chromosome group (8.9%, 13/147). Among the mutant signal patterns, 60 (68.2%, 60/88) were mutants of Oligo-pSc119.2-1, and 24 (27.3%, 24/88) were mutants of Oligo-pTa535-1, and in 4 (4.5%, 4/88) mutants, these two replaced each other. There were more Oligo-pSc119.2 mutants on the 2A and 7B chromosomes and more Oligo-pTa535 mutants on the 5D chromosomes than on the other chromosomes. Although there were only 27.3% signal pattern mutants of Oligo-pTa535-1, one point that cannot be ignored is that many Oligo-pTa535-1 signal patterns were dispersed on the chromosomes (Figure 1, Figure 2 and Figure 3). It was very difficult to distinguish the mutants, especially those with weaker signals. Therefore, the actual frequency of the mutants is most likely more than 27.3%.

### 2.5. ND-FISH Signals of the Tested Cultivars Compared with Those of Their Parents

To investigate the sources of the signal pattern polymorphisms, the ND-FISH signal patterns of five cultivars with clear and simple pedigrees were compared with those of their wheat parents.

**Figure 4:**
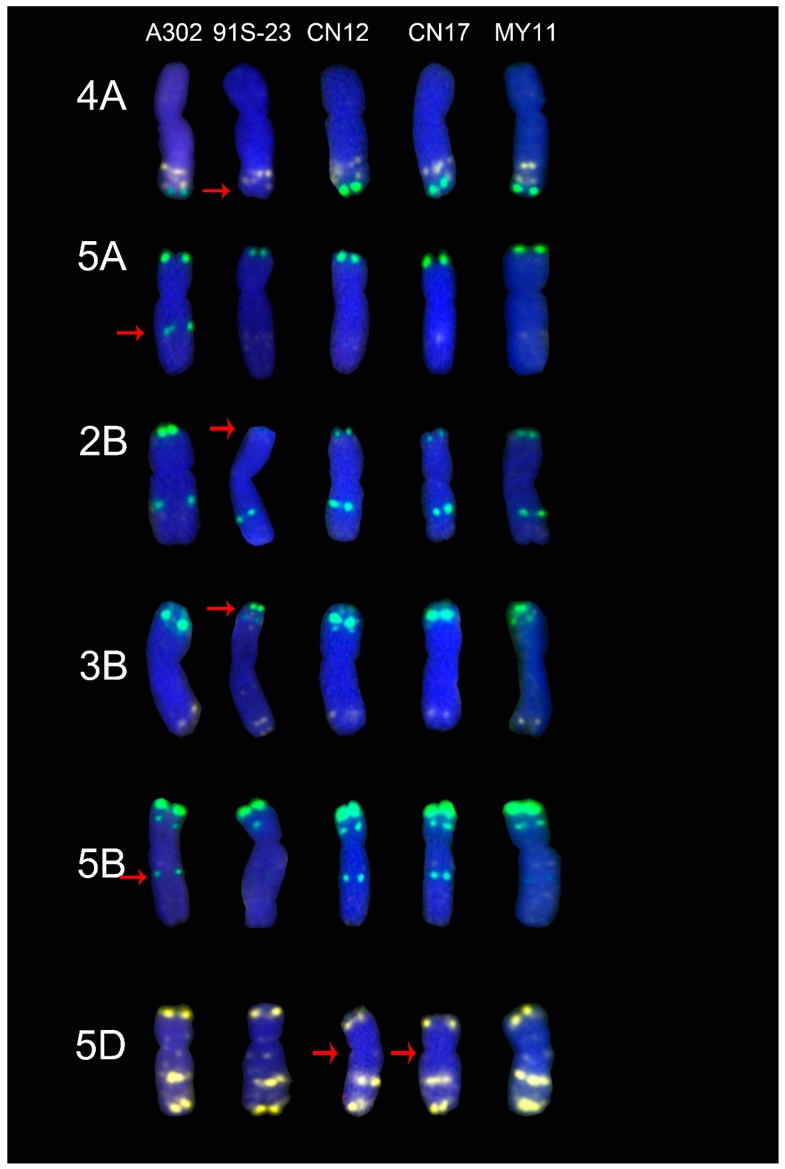
The differences of ND-FISH signal patterns in CN12 and CN17 cultivars when they were compared with their parents. From left to right: A302, 91S-23, CN12, CN17 and MY11, respectively. The red arrows showed the mutant signal patterns on chromosomes. Yellow: Oligo-pTa535-1; Green: Oligo-pSc119.2-1; Red: Oligo-Ku, Oligo-pSc200 and Oligo-pSc250. Blue: DAPI.

The CN12 and CN17 cultivars, which both originated from a cross of the A302 and 91S-23 cultivars, and their ND-FISH signals were compared with those of each other and their wheat parents (Figure 4). The A302 cultivar exhibited different ND-FISH signal patterns on the 5A and 5B arms. The 91S-23 cultivar showed different ND-FISH signal patterns on the 4A, 2B and 3B arms when they were compared with the standard patterns of the MY11 cultivar. Moreover, the signal from Oligo-pTa535-1 on the 5D centromeric region was missing in the CN12 and CN17 cultivars (Figure 4).

**Figure 5:**
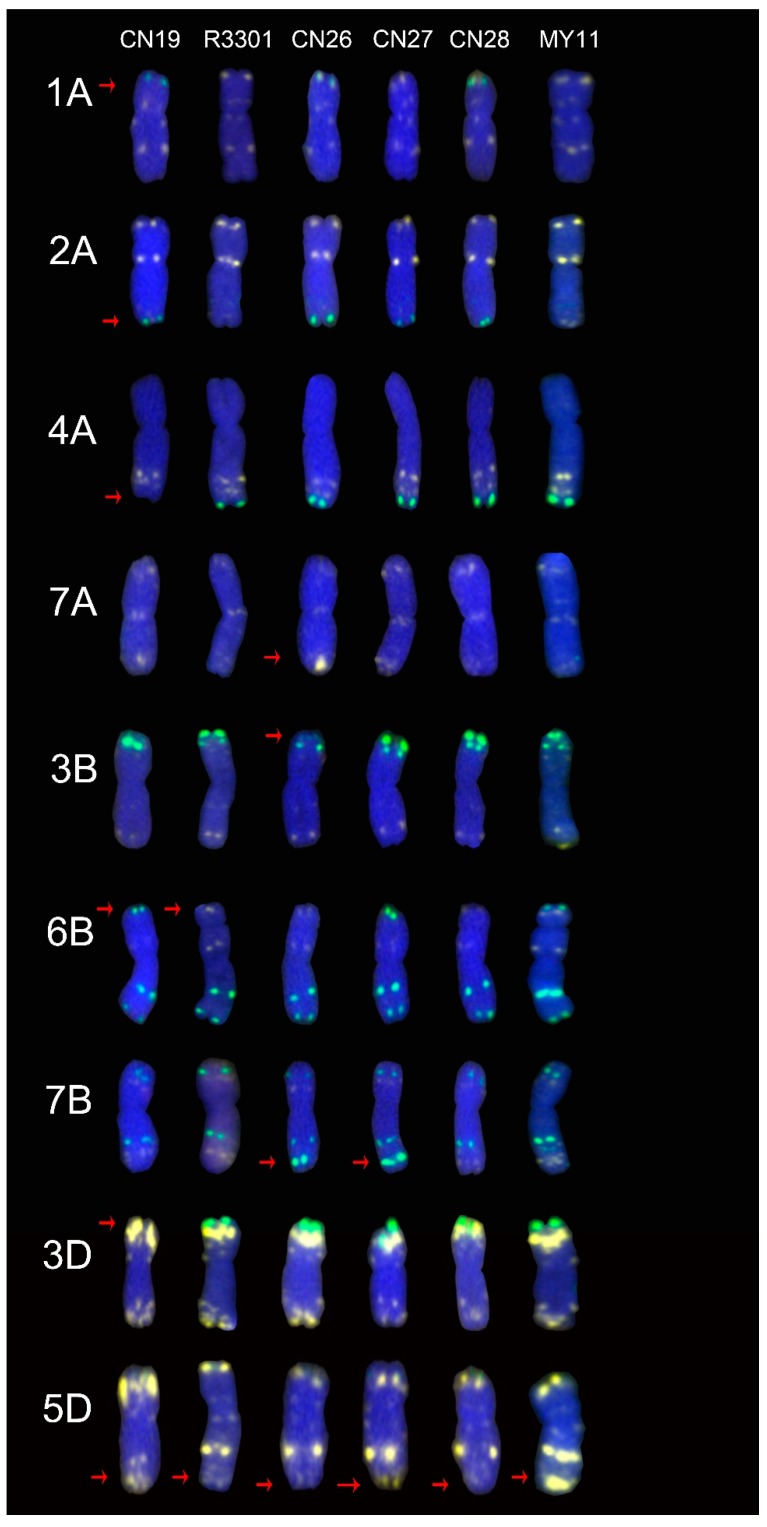
The differences of ND-FISH signal patterns in CN26, CN27 and CN28 cultivars when they were compared with their parents. From left to right: CN19, R3301, CN26, CN27, CN28 and MY11, respectively. The red arrows showed the mutant signal patterns on chromosomes. Yellow: Oligo-pTa535-1; Green: Oligo-pSc119.2-1; Red: Oligo-Ku, Oligo-pSc200 and Oligo-pSc250. Blue: DAPI.

The CN26, CN27 and CN28 cultivars, which all originated from a cross of CN19 and R3301 cultivars, were also analyzed. Their ND-FISH signal patterns were compared with those of their wheat parents and the MY11 cultivar (Figure 5). CN19 had different signals on the 1A, 2A, 4A, 6B, 3D and 5D arms when compared with the MY11 cultivar, and the R3301 cultivar had different signals on the 6B and 5D arms. The CN26 cultivar showed a strong signal from Oligo-pTa535-1 on the terminus of the 7A long arm, and the signal from Oligo-pSc119.2-1 was absent on the terminus of the 3B short arm (Figure 5). Novel signal patterns from Oligo-pSc119.2-1 were present on the 7B long arm in the CN26 and CN27 cultivars that were different than those of their parents and the MY11 cultivar. The strong signal from Oligo-pTa535-1 on the terminus of the 5D long arm was absent in CN19, R3301, CN26, CN27 and CN28, which was different than those observed in the MY11 cultivar (Figure 5).

## 3. Discussion

### 3.1. The Chromosome Structure of “Chuan-nong” Series Cultivars

The chromosome structure of the “Chuan-nong” series of cultivars was identified by ND-FISH. There were six 1RS.1BL translocation cultivars, all of which were released before 2003. CN10, CN11 and CN20 cultivars inherited their 1RS.1BL translocation chromosomes from the Aurora cultivar, in which the 1RS arm originated from the German rye Petkus cultivar. This cultivar harbors the *Yr9* and *Pm8* genes, which were resistant to stripe rust and powdery mildew. The CN12, CN17, and CN18 cultivars inherited their 1RS.1BL translocation chromosomes from the translocation line R14, whose 1RS arm originated from another German inbred rye line: L155; which harbors the *YrCn17* (resistant to stripe rust) and *PmCn17* (resistant to powdery mildew) resistance genes [23]. Unfortunately, the *Yr9* and *Pm8* resistance genes have lost their resistance since the 2000s in Southwestern China [23]. Furthermore, the resistance of *YrCn17* and *PmCn17* has weakened since 2010 [24], and the 1RS arm also has negative effects on grain processing quality [25,26,27]. Thus, when the 1RS chromosomes lose their resistance, they will be eliminated from the wheat breeding program. In this case, although the 1RS.1BL translocation chromosome is present in the pedigrees of most “Chuan-nong” series cultivars, released cultivars have not contained the 1RS.1BL translocation chromosome since 2004 (Table 1, Figure 2).

### 3.2. The Oligonucleotide Probes Can Replace the Use of Repetitive Sequence Probes

The pAs1 probe can distinguish the D-genome chromosomes of wheat [28], but Oligo-pTa535-1 can not only identify the group D chromosomes but also give rise to signals on the 1A, 2A, 3A, 4A, 6A, 7A, 3B, 6B and 7B chromosomes of wheat. It is better to use this oligonucleotide to distinguish wheat A-genome chromosomes because its signal in these chromosomes is strong and clear [9,18,29]. In ND-FISH, the oligonucleotide probe Oligo-pTa535-1 can replace the use of the repetitive sequence probe pAs1 [9].

The pSc119.2 probe can distinguish B-genome chromosomes and the 4A, 5A, 2D, 3D, and 4D chromosomes of wheat [9,17,28] and can also identify all rye chromosomes [5,30,31]. Oligo-pSc119.2-1 can distinguish B-genome chromosomes of wheat and rye, and this probe can also identify the 4A, 5A, 2D, 3D, and 4D chromosomes of common wheat [9,13,14,18,32]. These signals are similar to the pSc119.2 signal in B-genome chromosomes and rye chromosomes. Therefore, the Oligo-pSc119.2-1 oligonucleotide probe can also replace the use of the repetitive sequence probe pSc119.2.

The genomic DNA of rye is usually used as a probe to identify rye chromosomes in a wheat genetic background [3,5,23]. Fu et al. [13] reported that the combination of the oligonucleotide probes Oligo-1162, Oligo-pSc200 and Oligo-pSc250 identified rye chromosomes in a wheat genome. Li et al. [14] also reported that another combination of oligonucleotide probes (Oligo-KUD15, Oligo-pSc200 and Oligo-pSc250) distinguished the 1RS.1BL translocation chromosome and rye chromosomes in a wheat genetic background. A new oligonucleotide probe, Oligo-Ku, was recently developed. It can not only distinguish all rye chromosomes but also identify all *Dasypyrum villosum* chromosomes in the wheat genome [15]. In addition, similar to Oligo-KUD15, the signal patterns of Oligo-1162 and Oligo-Ku could not be observed on the wheat chromosomes [13,14,15]. Therefore, in ND-FISH, these oligonucleotide probes can replace the use of the genomic DNA of rye.

In the present study, the combination of Oligo-pSc119.2-1, Oligo-pTa535-1, Oligo-Ku, Oligo-pSc200 and Oligo-pSc250 was used to identify the chromosome structure of the “Chuan-nong” series of cultivars (Figure 1, Figure 2 and Figure 3). These newly developed oligonucleotide probes can perfectly replace the use of repetitive sequence probes such as pAs1 and pSc119.2. The combination of Oligo-pSc119.2-1 and Oligo-pTa535-1 can distinguish all 42 chromosomes of wheat, and the combination of Oligo-Ku, Oligo-pSc200 and Oligo-pSc250 can detect rye chromosomes in a wheat genetic background (Figure 2).

### 3.3. The Polymorphism Shown by ND-FISH Signal Patterns

Probes that were designed based on repetitive sequences sometimes display different signal patterns on the same chromosomes. Ren et al. reported that the signal patterns of pSc119.2 were different on the same chromosomes in different translocation lines [5,20]. ND-FISH provides a convenient and economical method for FISH analysis and very sensitive and reliable results for chromosomal mutants. However, the oligonucleotide probes that were developed from the same repetitive sequences displayed different signal patterns on the same chromosomes in different materials. Tang et al. [9] reported that Oligo-pSc119.2-1 and Oligo-pSc119.2-2 produced a signal on the 5AL arms of triticales but not on the 5AL arms of the parental wheat cultivar MY11. Jiang et al. [33] also reported that some oligonucleotide probes show different signal patterns on wheat chromosomes.

In the present study, Oligo-pSc119.2-1 and Oligo-pTa535-1 were used together to distinguish different wheat chromosomes. Most chromosomes showed the same signal patterns as the standard signal patterns of the MY11 cultivar. However, there were still several differences among them. These results indicate that oligonucleotide probes show high signal pattern polymorphism on wheat chromosomes. The polymorphism of the oligonucleotide probe signal patterns reflects differences in the distribution and copy numbers of tandem repeat sequences on chromosomes [5,15]. 

### 3.4. The Sources of the ND-FISH Signal Pattern Polymorphisms

To reveal the sources of the signal pattern polymorphisms, two groups of “Chuan-nong” wheat cultivars were compared with their wheat parents. Most of the mutant signal patterns from the cultivars were inherited from their wheat parents. For example, the signal pattern from Oligo-pSc119.2-1 on the 1A arm in CN26 and CN28 cultivars was inherited from the CN19 cultivar, but CN27 inherited the signal pattern from Oligo-pTa535-1 from the R3301 cultivar (Figure 5). This caused the polymorphism of the signal patterns on chromosome 1A. The source of this polymorphism is an independent assortment during crossing. In another case, the CN26 and CN27 cultivars showed different signal patterns on the 7BL arm when they were compared with their wheat parents and the MY11 cultivar (Figure 5). In addition, the signal pattern from Oligo-pTa535-1 on centromeric regions was different in CN12 and CN17 cultivars when they were compared with that of their parents and the MY11 cultivar (Figure 4). It was suggested that there are parallel repeat sequences of the oligonucleotide probes which were appeared or were absent after crossing. Feldman et al. [34] reported that DNA sequences could be silenced or eliminated during polyploidization. Tang et al. [35] also reported that polyploidization can induce mutations in tandem repeat sequences. Ren et al. [5,20] reported that the signal pattern from pSc119.2 was mutated after chromosome translocation. There have been no reports of rapid mutations due to crossing. In the present study, CN19 was shown to be a normal wheat cultivar without any rye chromatin, but R3301 was a 1RS.1BL translocation line. The cross between CN19 and R3301 could cause monosomy of the 1RS.1BL translocation chromosome and the 1B chromosome. Monosomy usually leads to the instability of the genome [36,37]. Therefore, the effects of monosomy could explain the mutations in the CN26 and CN27 cultivars. Both 91S-23 and A302 were shown to be 1RS.1BL translocation lines, so a cross between them would not form a monosomic line. However, the progenies of this cross; the CN12 and CN17 cultivars, also showed different signal patterns. This suggests that a normal cross between wheat can also result in DNA mutations.

## 4. Materials and Methods

### 4.1. Plant Materials

The “Chuan-nong” series of wheat cultivars have been the most important and widely used in southwestern China since the 2000s. These cultivars show many specific agronomic traits, such as staying green [38], high tiller numbers [39], high yield [24,40], and great resistance [23,41,42].

Eighteen released “Chuan-nong” series of wheat cultivars, 3 new lines, and several of their wheat parents were used for the study (Table 1). The common wheat (*Triticum aestivum* L.) cultivar MY11, which was a standard ND-FISH signal pattern, was used as a control [9]. All plant materials were provided by the Provincial Key Laboratory of Plant Breeding and Genetics of Sichuan Agriculture University.

### 4.2. Identification of Chromosomes

All plant materials were identified by non-denaturing fluorescence in situ hybridization (ND-FISH). Five probes (Oligo-pSc119.2-1, Oligo-pTa535-1, Oligo-Ku, Oligo-pSc200 and Oligo-pSc250) were mixed and used on one slide. The sequences of the probes are listed in Table 2 [9,15].

All probes were synthesized by Tsingke Biological Technology Co. Ltd. (Beijing, China). The Oligo-Ku probe was 5′-end labeled with 6-carboxytetramethylrhodamine (TAMRA), and its signal patterns are displayed in red. The Oligo-pSc119.2-1 probe was 5′-end labeled with 6-carboxyfluorescein (6-FAM), and its signal patterns are displayed in green. The Oligo-pTa535-1 probe was 5′-end labeled with cyanine 5 (Cy5), and its signal patterns are displayed in yellow. The Oligo-pSc200 and Oligo-pSc250 probes were 5′-end labeled with 6-carboxytetramethylrhodamine (TAMRA), and their signal patterns are also displayed in red. Chromosomes were counterstained with 4′,6-diamidino-2-phenylindole (DAPI) and are displayed in blue. Non-denaturing fluorescence in situ hybridization and image capture were conducted according to Tang et al. [9], Li et al. [14] and Ren et al. [5]. For each plant material, ND-FISH was repeated three times.

## Figures and Tables

**Table 1:** The pedigree of “Chuan-nong” series wheat cultivars.

Cultivars	Cross Combinations	Released Time
Chuan-nong10 (CN10)	78-5038 ^a^ × 85DH5015	2003
Chuan-nong11 (CN11)	78-5038 ^a^ × 85DH5015	2001
Chuan-nong12 (CN12)	91S-23 ^b^ × A302	2002
Chuan-nong17 (CN17)	91S-23 ^b^ × A302	2002
Chuan-nong18 (CN18)	R164-1 ^b^ × A302	2003
Chuan-nong19 (CN19)	Qian1104A × R935 ^b^	2003
Chuan-nong20 (CN20)	78-5038 ^a^ × 85DH5015	2003
Chuan-nong21 (CN21)	R841 ^b^ × Qianhui3	2004
Chuan-nong22 (CN22)	R164 ^b^ × 86-104	2005
Chuan-nong23 (CN23)	R1685 ^b^ × MY26	2005
Chuan-nong24 (CN24)	CN10 ^a^ × Yunfan52894-2	2007
Chuan-nong25 (CN25)	96 I-225 × 91S-5-4 ^b^	2007
Chuan-nong26 (CN26)	CN19 × R3301 ^b^	2006
Chuan-nong27 (CN27)	CN19 × R3301 ^b^	2009
Chuan-nong28 (CN28)	CN19 × R3301 ^b^	2011
Chuan-nong29 (CN29)	(02017 × CN19) × R131	2015
Chuan-nong30 (CN30)	(03FR1349-1 × 54789) × CN27	2016
Chuan-nong31 (CN31)		Not release
Chuan-nong32 (CN32)	CN27 × 80978	2017
Chuan-nong33(CN33)		Not release
Chuan-nong35 (CN35)		Not release
Mianyang11(MY11)	control	

Superscript “a” indicated the plant material harboring 1RS.1BL translocation chromosome from Aurora. Superscript “b” indicated the plant material harboring 1RS.1BL translocation chromosome from R14, which bring resistance gene *YrCn17* and *PmCn17* [22].

**Table 2:** The nucleotide sequences of oligonucleotide probes.

Probes	Sequences (5′-3′)
Oligo-pSc119.2-1	CCGTTTTGTGGACTATTACTCACCGCTTTGGGGTCCCATAGCTAT
Oligo-pTa535-1	AAAAACTTGACGCACGTCACGTACAAATTGGACAAACTCTTTCGGAGTATCAGGGTTTC
Oligo-Ku	GATCGAGACTTCTAGCAATAGGCAAAAATAGTAATGGTATCCGGGTTCG
Oligo-pSc200	CTCACTTGCTTTGAGAGTCTCGATCAATTCGGACTCTAGGTTGATTTTTGTATTTTCT
Oligo-pSc250	TGTGTTGTTCTTGGACAAAACAATGCATACCATCTCTTCTAC
